# Reduction of Calcium Scores Using Intravenous Chelation: A Retrospective Pilot Study

**DOI:** 10.7759/cureus.44657

**Published:** 2023-09-04

**Authors:** Stephen J Petteruti, Vincent Frazzini

**Affiliations:** 1 Family Medicine, Kent County Memorial Hospital, Warwick, USA; 2 Radiology, Tollgate Radiology, Warwick, USA

**Keywords:** coronary artery disease, preventative cardiology, noninvasive cardiac testing, coronary artery calcium, calcium score, chelation therapy, statins, coronary heart disease, coronary artery calcification, atherosclerotic cardiovascular disease

## Abstract

This pilot study presents a retrospective analysis of 10 asymptomatic patients with a positive calcium score who received a series of intravenous calcium ethylenediaminetetraacetic acid (EDTA) chelations. Current standards for cardiovascular risk stratification include assessments of cholesterol, blood pressure, blood sugar, lifestyle, obesity, and family history. Despite addressing traditional risk factors, myocardial infarctions and cerebrovascular accidents remain the leading causes of death and disability worldwide. Asymptomatic decay of the vascular system is a prelude to catastrophic events, and calcium scores are emerging as a significant adjunct for risk assessment. Positive calcium scores correlate with an increased risk of cardiovascular events. However, there are no therapies known to reliably reverse calcium scores. Previous studies have demonstrated that intravenous chelation therapy reduces cardiovascular morbidity and mortality in patients with a prior history of myocardial infarction; however, its mechanism of action is unknown. One theory is that chelation therapy would reverse calcium buildup in coronary arteries, which is known to have a positive correlation with the risk of having a cardiovascular event.

The 10 patients had no prior history of coronary artery disease. Infusions were administered in an outpatient setting. Patients were encouraged to receive a treatment every month. No other supplements or prescriptions were required as part of the treatment. An average of 26.9 chelations were administered over an average of 37.9 months. Calcium scores decreased by an average of 27.38%, and all 10 patients experienced a reduction in scores.

This study demonstrates that chelation has the potential to reduce calcium scores. Since calcium scores correlate with cardiovascular risk, reducing the calcium score may reduce the risk of an event. If these results are supported by larger, placebo-controlled studies, chelation therapy may become an option that could be added to statins and other FDA-approved therapies for primary prevention in patients with a positive calcium score.

## Introduction

Treatment to lower cholesterol has been a cornerstone of cardiac risk reduction for decades. However, studies of one drug (Evacetrapib) did not demonstrate any benefit regarding cardiac risk reduction despite having a profound effect on lowering cholesterol [1]. Although statin drug therapy has had a beneficial effect on coronary artery disease, it is possible that we have reached the limit of what can be achieved with lipid-lowering therapy. The need for new methods for evaluating and reducing cardiac risk is apparent.

Calcium scores have emerged as a powerful tool for assessing the risk of future cardiac events [2]. Even in individuals who have risk factors for heart disease, a calcium score of "0" has a powerful negative predictive value regarding future events [3]. In addition, calcium score progression has been correlated with an increased risk of subsequent cardiac events [4-6]. Despite the value of the information, the role of calcium scores for screening and following disease progression remains uncertain. Some of the uncertainty may come from the fact that there is no consensus on how to treat a positive calcium score [7].

Despite the strong evidence that statin therapy reduces cardiac events and the growing evidence that statin therapy can reduce soft plaque buildup, a study on the effect of statin therapy when compared with placebo did not show any significant impact on calcium score progression [8]. In another study, there was no difference between low-dose (10 mg) and high-dose (80 mg) atorvastatin therapy. Both groups had a progression of the calcium score [9]. Therefore, if the prevailing evidence is that calcium scores will progress regardless of therapy, it is reasonable to forgo the calcium score altogether for the purpose of sequentially monitoring cardiac risk.

A previous study has demonstrated a positive correlation between chelation therapy and the reduction of myocardial infarctions, cerebrovascular accidents, and other events; however, the mechanism of action for chelation has not been defined. It is thought that the benefits may be mediated by the reduction of metals, especially lead and cadmium, which are both linked to cardiovascular risk. In addition, it has been theorized that chelation may reduce reactive oxygen species by interfering with the metals that bind to glycation end products. Oxidized end products accumulate in the tissue and promote inflammation. Oxidative stress is felt to be a major contributor to atherosclerosis [10].

Intermittent chelation therapy has also demonstrated the capacity to reduce total body lead levels [11]. These two studies provide supporting evidence that intravenous chelation therapy may reduce both cardiac events and accumulated lead levels. Research suggests that one of the mechanisms of action for cardiac benefit may be the reduction of total body lead levels, which is known to be toxic to the heart and arteries [12,13].

The current retrospective observational cohort was undertaken to assess the impact of long-term, intermittent intravenous chelation therapy with calcium ethylenediaminetetraacetic acid (EDTA) and other micronutrients on calcium scores.

## Materials and methods

Methods

This was a retrospective observational cohort study made up of 10 patients who were from the primary care private practice of the lead author. As part of the practice's focus on cardiovascular disease prevention, calcium scores are offered to patients aged 50 and above or those at a younger age with cardiac risk factors. Chelation therapy was offered based on its theoretical potential to reduce coronary artery disease risk and affect calcium score progression. Statin therapy and other means of risk reduction (FDA-approved medications, lifestyle, and weight management) are also offered. In addition to having a positive calcium score, the inclusion criteria included the absence of pre-existing cardiovascular disease as assessed by clinical history, a physical examination, and a normal basic metabolic profile. The criteria for exclusion were a creatinine greater than 1.2. The primary outcome was a change in calcium score. 

The aim of the study was to test the hypothesis that a series of intravenous chelation infusions would reduce the patient’s calcium score. Written informed consent was obtained from all study participants. Patients received an initial calcium score, followed by a series of intravenous chelation treatments, and then had a follow-up calcium score three years later. None of the patients had active cardiopulmonary symptoms. None of the patients had a prior history of myocardial infarction or any known cardiovascular disease. None had a history of a prior calcium score.

All calcium scores were performed at the same location and with the same equipment. All calcium scores were interpreted using the same computer program in addition to being read by the same radiologist. The radiologist had no knowledge regarding the chelation status of the patients. Patients were then advised to undergo intravenous chelation therapy monthly using one of two chelation formulas. The decision regarding which formula to use was based on the clinical judgment of the provider. 

Calcium scores are reported by the “Agatston” scoring method. The Agatston score is a mathematical calculation that is utilized for the purpose of enabling comparisons between calcium scores that are collected on different equipment.

Materials 

Calcium scores were performed on a Neusoft 64 CT with a slice thickness of 2.5 mm and a scan interval of 10.0 mm. Each calcium score was computer analyzed and interpreted by one of the authors, a board-certified radiologist.

Infusion “A”

Infusion A consists of the following ingredients: calcium EDTA at a calculated dose of no greater than 3,000 mg, vitamin C at 7,500 mg, magnesium at 1,500 mg, vitamin B12 at 1,000 µg, and B complex vitamins at 1 mL.

Infusion "B"

Infusion B consists of the following ingredients: calcium EDTA at 900 mg, vitamin C at 2,000 mg, L-arginine at 500 mg, magnesium chloride at 400 mg, vitamin B12 at 1,000 µg, B complex vitamins at 1 mL, l-carnitine at 500 mg, folic acid at 10 mg, biotin at 10 mg, and glutathione given as a separate push at 600 mg.

In each case, ingredients were mixed in 250 mL of normal saline or sterile water and infused over approximately one hour.

## Results

Ten patients were evaluated as part of the study. The median number of infusions performed per patient was 25 (Interquartile Range = 13). The infusions were performed over an average period of 37.9 months (Interquartile Range = 11), which works out to approximately one infusion every 1.4 months.

All subjects were Caucasian. Seven of the 10 were male, and the other three were female. Five of the patients had a diagnosis of hypertension, one had a diagnosis of diabetes, and three were obese. None of the patients were current cigarette smokers. Nine of the 10 clients were receiving testosterone cypionate as part of their hormone replacement therapy, including all three female patients. The use of testosterone therapy reflects the practice's focus on treating patients who have testicular hypofunction, erectile difficulty, and/or decreased libido. Testosterone supplementation has not been shown to increase CV events and was not a component of the criteria for obtaining calcium scores or receiving chelation therapy.

**Figure 1:**
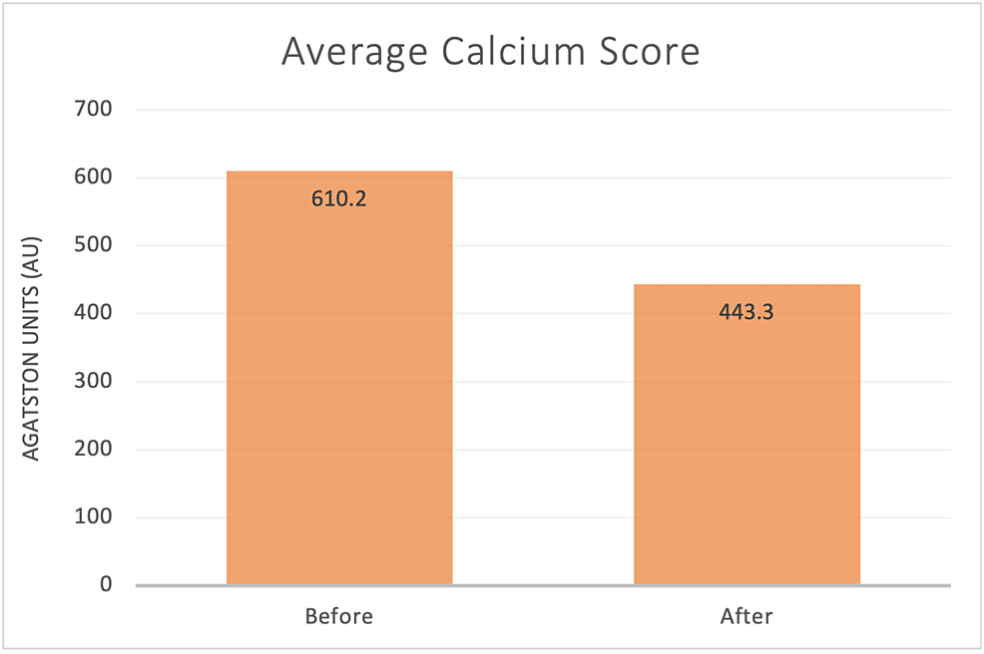
Average Calcium Score Before and After Chelation Therapy The bar graph represents the average calcium score, in Agatston units, for all 10 patients before and after their chelation therapy. All 10 patients observed a reduction in calcium score, with an average decrease of 27.38%.

All 10 of the patients had a reduction in their calcium scores. The average calcium score at the outset was 610.2 Agatston units, whereas the average follow-up calcium score at the end of the treatment cycle was 443.3, for an average reduction of 27.38%. (If patient #3 is excluded as an outlier, the average reduction is 23.77%). The range of reduction in percentage was from 7.14% to 88.33%. Three of the patients only received chelation infusion A. Those patients had the following changes: one patient had an initial score of 98 and a second score of 91, for a decrease of 7.14%. The second patient had an initial score of 1049 and a second score of 883, for a reduction of 15.82%. The third patient had an initial score of 470 and a second score of 347, for a reduction of 26.17%. One patient received only the chelation infusion B. This patient had a reduction in calcium score from 426 to 297, for a reduction of 30.28%, after a series of 20 infusions over a period of 37.12 months. There were no adverse events noted in any of the patients. None of the patients experienced any medical events. There were no myocardial infarctions, cerebrovascular accidents, hospitalizations, or other medical problems over the span of therapy. Table 1 summarizes the results for each patient, and Table 2 provides pertinent averages. Table 3 summarizes relevant clinical information on each patient. Table 4 lists relevant lab values. Figure 1 is a bar graph representing the average calcium score before and after chelation.

**Table 1:** Summary of Results “A” = Infusion A. “B” = Infusion B. Total = total infusions. Months = duration of treatment.

Patient	Age	Date	First Score	Date	Second Score	% Change	1^st^IV	2^nd^IV	“A”	“B”	Total	Months	IV/Month
1	51	Aug, 2018	605	Jun, 2020	516	-14.88	Jun, 2018	Jun, 2020	15	4	19	24	1.26
2	67	Jan, 2017	781	Jun, 2020	555	-28.29	Jan, 2017	Apr, 2020	23	2	25	39	1.56
3	56	Sep, 2015	6	Jul, 2019	1	-83.33	Oct, 2015	Jul, 2019	31	2	33	45	1.36
4	52	Apr, 2018	98	May, 2020	91	-7.14	Jun, 2018	May, 2020	25	0	25	22	0.88
5	71	Nov, 2016	220	Jul, 2020	97	-55.9	Mar, 2017	Jul, 2020	29	7	36	40	1.11
6	59	Sep, 2016	1049	Aug, 2020	883	-15.82	Sep, 2016	Aug, 2020	25	0	25	47	1.88
7	57	Feb, 2017	1668	Sep, 2020	1164	-30.22	Feb, 2017	Aug, 2020	3	29	32	42	1.31
8	70	Sep, 2016	779	Sep, 2020	482	-38.13	Sep, 2016	Sep, 2020	18	17	35	48	1.37
9	73	July, 2017	470	Sep, 2020	347	-26.17	Jul, 2017	Sep, 2020	20	0	20	38	1.90
10	86	Apr, 2018	426	Feb, 2021	297	-30.28	Apr, 2018	Feb, 2021	0	19	19	34	1.79

**Table 2:** Patient Averages

	Age	First Score	Second Score	% Change	Total	Months	IV/Month
Average	64.2	610.2	443.3	-27.38	26.9	37.9	1.4
Median	59	470	297	-28	25	38	1.31

**Table 3:** Clinical Information for Patients BMI = body mass index. BP Meds = blood pressure medication. DM 2 = diabetes mellitus type 2. CAD = coronary artery disease. HTN = hypertension

Patient	Age	Sex	BMI	DM 2	HTN	CAD	Statin Use	BP Meds
1	51	M	33.7	NO	YES	NO	NO	YES
2	67	M	27.1	NO	NO	NO	YES	NO
3	56	M	27.3	NO	NO	NO	NO	NO
4	52	M	50.3	YES	YES	NO	NO	NO
5	71	M	26.8	NO	NO	NO	NO	NO
6	59	M	31.4	NO	NO	NO	NO	NO
7	57	F	23.6	NO	YES	NO	NO	YES
8	70	F	24.5	NO	YES	NO	NO	YES
9	79	M	23.2	NO	NO	NO	NO	NO
10	86	F	27.4	NO	YES	NO	NO	YES

**Table 4:** Lab Values at Start of Chelation Therapy A1C = Hgb A1C. HDL = high-density lipoprotein. HS CRP = high-sensitivity C-reactive protein. LDL = low-density lipoprotein. NA = result not available.

Patient	Cholesterol mg/dL	Triglyceride mg/dL	HLDL mg/dL	LDL mg/dL	A1C%	Creatinine mg/dL	Homocyst	HS CRP
1	228	175	37	156	5.3	0.87	11.2	1.2
2	199	103	49	129	5.3	1.11	10.6	0.5
3	253	109	46	199	5.3	1.09	5	1.2
4	151	245	36	66	6.8	0.88	6.4	9.3
5	179	80	63	100	5.7	1.05	21.1	11.1
6	151	186	32	82	4.6	1.19	10.3	0.3
7	235	160	94	109	5.2	0.75	NA	NA
8	360	142	49	283	5.5	0.98	9.8	3.4
9	181	57	52	118	NA	0.9	NA	NA
10	145	84	56	72	5.3	0.62	NA	1

## Discussion

Calcium scores have been recognized as a valuable predictor of future cardiovascular events [2,3,14,15]. Despite their usefulness in forecasting future events, they have not been widely utilized for routine screening. It is possible that part of the reason for the failure to adapt this simple screening exam is that there is no consensus on how to treat a positive result [7]. There are pharmacologic options to address high blood pressure and elevated cholesterol, but thus far, there has been no reliable intervention proven to consistently reduce calcium scores.

Studies investigating the use of statin drugs have been disappointing regarding their impact on calcium scores. The PARADIGM study evaluated individuals who were on statins versus those who were not and compared their baseline and follow-up calcium scores. The average time interval from the first study to the follow-up was approximately 3.9 years. Both groups had an increase in calcium scores [15]. This result, along with those of other studies, has led to the widely held presumption that a calcium score, once established, will inevitably progress regardless of the intervention. Therefore, if there is no treatment of consequence and if all calcium scores eventually progress, it is understandable why there is little enthusiasm for ordering a study for which there is no reliable treatment [16].

Up until this point, the impact of IV chelation therapy on calcium scores has not been documented. This study was motivated by the theory that for chelation therapy to reverse calcium scores, it would require a long duration of treatment spanning years. The recommendation for monthly intervals of chelation was arrived at based in part on practical considerations. Because chelation therapy is not insurance-reimbursed, any use of it for the treatment of calcium scores must be logistically and financially practical; otherwise, the results would not have clinical value. In addition, a previous study demonstrated that monthly intervals of chelation therapy extended over a prolonged period can be effective at lowering total body levels of lead [11]. This established a basis for the frequency of chelation. Although this was a pilot study, the results are nevertheless impressive. All 10 of the subjects had a reduction in their calcium score. The reductions cut across all ages and included a subject with diabetes and morbid obesity. The reduction occurred in both men and women, in patients of advanced age, and in patients with high and low initial scores. 

An advantage of this study is the fact that the results are purely objective. There was no role for interpretive bias or patient-reported variations. The same CT scanner was used for the pre-and-post studies, and the same computer-generated programs were used to calculate the calcium scores.

Although the study was neither designed nor powered to assess definitive clinical outcomes such as myocardial infarction (MI), cerebrovascular accident (CVA), or death, it is noteworthy that there were no cardiovascular events throughout the course of the study. In addition, there was no change from baseline creatinine levels, and no subjective or objective complications were reported.

Only one of the participants was on statin drug therapy. This was not by design but a consequence of patient choice. The patients in the study were more accepting of intermittent infusions of chelation therapy than they were of taking a daily oral drug. Even though most in this cohort chose not to use statin therapy, there is no need to refrain from using statin drug treatment in the proper setting. Although study results have been disappointing regarding the reversal of calcium scores with statin drug treatment, there is evidence that the drugs diminish the soft plaque, which cannot be seen in calcium scores but is a contributor to MI and CVA [15].

Limitations

This was a small retrospective pilot study, and the subjects acted as their own controls. There was no placebo group. The group also lacked ethnic diversity. The fact that there were two different chelation protocols utilized further limits the interpretation. Although the objective results are noteworthy (all 10 of the subjects having a reduction in calcium score), we must consider a 100% response to any therapeutic intervention as unlikely. Also, since this was a clinical retrospective observation, the duration of treatment and the frequency of treatment intervals varied among the patients. 

All the patients in the study were in good health and had no acute medical conditions requiring intervention. Their primary motive was the maintenance of health and the desire for preventative treatments to diminish the risk of chronic disease. The patients included in the study are proactive about pursuing a healthy lifestyle. They are all physically active, thoughtful with nutrition, and non-smokers. This presents the potential for a selection bias amongst the cohort that makes the results difficult to generalize.

Although the possibility of observer bias always needs to be considered, it should be noted that calcium score results are computer-generated and that the same CT machine and software were used for all the studies, thus further limiting the risk of bias. The role of the radiologist is to review the computerized results and add context to the interpretation.

## Conclusions

This study demonstrates that IV chelation therapy has the potential to reduce calcium scores. Although elevated calcium scores are a recognized risk factor for coronary artery disease, it remains to be determined whether or not reducing calcium scores by virtue of chelation therapy will have any impact on morbidity or mortality. In addition, although there was a reduction in calcium scores in the study, the post-treatment calcium scores were still in a range considered to be consistent with an elevated risk of subsequent cardiac events. It is also unclear which aspect of the infusion was responsible for the reduction in calcium scores. Lead is considered to be a cardiotoxin, and calcium EDTA has been demonstrated to reduce lead levels. However, these infusions also had significant antioxidants in them. Antioxidant therapy has been theorized to have value in reducing the inflammatory effect on coronary arteries. Therefore, the ingredients responsible for the reduction in the calcium score remain undetermined.

Given the fact that cardiac disease remains the leading cause of death in developed nations despite the widespread use of antihypertensive and cholesterol-lowering drug therapies, additional approaches to reduce risk are needed. Chelation therapy has demonstrated the capacity to reduce cardiac events in individuals with a history of a prior MI and has been shown to be low-risk. This study adds information regarding the macro-vascular and anatomical effects of chelation therapy. Based on the results of our study, we recommend that if individuals choose to add chelation as part of their response to an elevated calcium score, they should receive it at monthly intervals. They should also wait at least three years to perform a follow-up calcium score to assess the impact of therapy. Furthermore, patients should be advised that chelation therapy is not an FDA-approved treatment for the prevention of heart disease and that the benefit of chelation therapy in preventing cardiac events remains undetermined. Chelation therapy should not be used in place of currently established, FDA-approved pharmacotherapies such as statin drugs and antihypertensive agents.
